# Understanding COVID-19 vaccine hesitancy in vasculitis patients

**DOI:** 10.3389/fpubh.2023.1301492

**Published:** 2023-12-04

**Authors:** Imama N. Butt, Charmaine van Eeden, Katharina Kovacs Burns, Lynora Saxinger, Alison Clifford, Desiree Redmond, Jan Willem Cohen Tervaert, Elaine Yacyshyn

**Affiliations:** Department of Medicine, University of Alberta, Edmonton, AB, Canada

**Keywords:** vaccine hesitancy, COVID-19 vaccines, vasculitis, patient perceptions, SARS CoV-2

## Abstract

**Objective:**

To identify the factors that impact COVID-19 vaccine decision-making in vaccine-hesitant vasculitis patients, and compare their perceptions with other rheumatology patients, given existence of data suggesting rheumatology patients may have disease-specific factors that influence their COVID-19 vaccine decision-making.

**Methods:**

This cross-sectional study surveyed adult rheumatology patients from the Kaye Edmonton Clinic Rheumatology Clinic, in Canada, between June and August 2021, using an anonymous online questionnaire. Survey responses were analyzed for statistical differences using chi-square analysis.

**Results:**

The COVID-19 Vaccine Perceptions Survey had a response rate of 70.9%. Of the total 231 respondents, 103 patients were diagnosed with vasculitis. At the time of the survey, 10.6% of vasculitis patients refused to receive a COVID-19 vaccine compared to 6.3% for other rheumatology patients. Compared to other rheumatology patients, vaccine-hesitant vasculitis patients were significantly more concerned about almost every aspect of available COVID-19 vaccines [e.g., safety (*p* < 0.001), components (*p* < 0.001)], and feared that they could contract SARS-CoV-2 from a vaccine (*p* < 0.001). These vaccine-hesitant patients were also significantly less pleased with the government's pandemic response, less confident in healthcare team-provided information (*p* < 0.001), and more likely to report that healthcare providers had no role in their COVID-19 vaccine decision-making (*p* < 0.001).

**Conclusion:**

Vaccine-hesitant vasculitis patients may have multiple considerations influencing COVID-19 vaccine hesitancy, including vaccine and disease-specific concerns, along with unfavorable perceptions of the healthcare system (government and healthcare providers). Healthcare providers can address some of these concerns by initiating patient-centered discussions around immunizations to help support educated decision-making.

## 1 Introduction

Vaccination against SARS-CoV-2 is an important tool in the management of the COVID-19 pandemic (1). It is critical to optimize vaccination uptake amongst the public, particularly in vulnerable populations, at greater risk for contracting infectious diseases, including those with rheumatic conditions, associated comorbidities, and therapies used for disease management (2). Relatedly, some rheumatology patients can be at greater risk of contracting SARS-CoV-2 (3, 4), and some data suggests increased risk of poor outcomes after developing COVID-19 (3–6). Specifically, vasculitis patients are at a potentially elevated risk, as preliminary data suggests that these individuals can develop severe COVID-19 disease, with high morbidity and mortality due to underlying conditions and treatments, compared to the general public (7, 8). Additionally, patients with vasculitis have reported stronger negative beliefs about their disease and its implications on their emotional and physical wellbeing compared with other chronic illnesses (9). Regarding the COVID-19 pandemic, patients with vasculitis described high concerns about the pandemic, because of their underlying vasculitis diagnosis (10). Despite increased concerns, many vasculitis patients engaged in harmful health-related behaviors, such as avoiding doctors' office visits, laboratory tests, along with stopping or delaying immunosuppressive medications, often without consulting their healthcare providers (10). Therefore, vaccination against SARS CoV-2 is an important tool to help manage the substantial risk that COVID-19 poses to vasculitis patients' overall health.

Previous international studies have demonstrated significant vaccine hesitancy in rheumatology patients (11, 12). Recent studies indicated that patients with autoimmune and inflammatory rheumatic diseases (AIIRDs), undergoing treatment, were less likely to be vaccinated (76.9 vs. 87.0%) (13), and 32% less likely to receive a COVID-19 vaccine, compared to non-AIIRDs patients (14). Multiple factors could be contributory, but some vasculitis patients are noted to have specific concerns that vaccines may have triggered or exacerbated their autoimmune condition (15, 16). However, recent systematic reviews show limited evidence to support *de novo* systemic vasculitis post-COVID-19 vaccination (17). Although there are case reports of small-vessel vasculitis as an adverse reaction to SARS-CoV-2 immunization, this is a rare phenomenon, and is typically cutaneous—or renally—limited, transient, with good prognosis (17). Finally, while additional knowledge is emerging, initial COVID-19 vaccine clinical trials excluded immunocompromised patients, which could contribute to rheumatology patient reluctance because of uncertainty around safety or effectiveness of the vaccines (18).

The above factors, along with vaccine misinformation and disinformation, challenge the success of COVID-19 immunization programs (19), emphasizing the need to understand vaccine-hesitant rheumatology patients' perceptions. This knowledge can help healthcare providers in discussions with patients to alleviate concerns, support educated medical decision-making, and potentially encourage vaccine uptake.

The objective of this study was to identify the factors that impact COVID-19 vaccine decision-making in vaccine-hesitant vasculitis patients compared with other rheumatology patients, given that vasculitis patients demonstrated increased COVID-19 related concerns and harmful health-related behaviors, which could exacerbate their pre-existing risk for contracting and developing poor outcomes from SARS CoV-2.

## 2 Materials and methods

### 2.1 Study design

This cross-sectional descriptive survey aimed to explore perceptions of COVID-19 vaccines among rheumatology patients. This study compared factors that influence vaccine decision-making in a sub-group of patients diagnosed with vasculitis, compared to other rheumatology patients. Anonymous participants responded to an online questionnaire using the REDCap platform, between June and August 2021.

### 2.2 Study setting

This study was conducted at the Kaye Edmonton Clinic (KEC) Rheumatology Clinic, in Edmonton, Alberta, Canada. Patients seen in the Rheumatology Clinic are by referral only, and include individuals living anywhere in Edmonton, and surrounding areas.

### 2.3 Study participants

Patients were sequentially recruited from a convenience sample of adult (≥18 years) rheumatology patients (diagnosed with one or more rheumatologic condition) seen in-clinic at the KEC between June and August 2021. All participants were recruited from the same physician clinics. Potential participants were informed of the research study and its purpose when they attended a scheduled appointment. Interested patients voluntarily provided their email address and were subsequently forwarded a link to an anonymous online survey. Participants were required to have their own device with reliable internet access. Participants were informed that they could respond to some or all questions and could also withdraw entirely by not submitting the survey.

Vasculitis patients made up 45% of all rheumatology patient participants, permitting relatively similar sample sizes for analysis and comparison. Rheumatic conditions included spondyloarthropathies (ankylosing spondylitis, psoriatic arthritis), rheumatoid arthritis, fibromyalgia, gout, lupus, myositis, scleroderma, polymyalgia rheumatica (PMR), sarcoidosis, tendonitis/bursitis, and osteoporosis. Vasculitis diagnoses included large vessel vasculitis (Giant Cell Arteritis, Takayasu's arteritis), ANCA-associated vasculitis (GPA, MPA, EGPA), and small vessel vasculitis (IgA vasculitis).

### 2.4 Survey development

The COVID-19 Vaccine Perceptions Survey has been previously described (20), and was a 44-item online REDCap survey, that included quantitative and qualitative items. The survey was based on a review of vaccine hesitancy literature as well as circumstances and messaging regarding vaccination at the time (11, 12, 21). To identify factors that could impact decision to vaccinate, the survey included demographics (22–25), patient self-reported medical condition(s), and treatment, as well as views around contracting SARS-CoV-2, COVID-19 vaccine concerns, views of the government's role in handling the COVID-19 pandemic (26), and questions regarding informed decision-making. As previous work suggested the influence of healthcare teams in promoting vaccine acceptance (27–29), the final survey section also asked participants about their perceptions of their healthcare team. The survey was pilot tested for a grade eight reading and comprehension level. Patients were provided a unique link to the online survey, determined to take 20 min to complete based on pilot-testing.

### 2.5 Data analysis

All quantitative questions were descriptively analyzed (i.e., percentages and frequencies) along with chi-square analysis to determine statistical significance (*p* < 0.05) using STATA 17 (StataCorp. 2021. Stata Statistical Software: Release 17. College Station, TX: StataCorp LLC). Vaccine-hesitant vasculitis patients were defined as those who indicated that they had been diagnosed with vasculitis and stated that they did not want a COVID-19 vaccine at the time of the survey. Responses from the vaccine-hesitant vasculitis patients were analyzed separately and compared to other non-vaccine-hesitant vasculitis responders.

### 2.6 Ethics approval

This study received ethics approval from the Health Research Ethics Board at the University of Alberta (Pro00108774).

## 3 Results

The COVID-19 Vaccine Perceptions Survey had a response rate of 70.9% across all rheumatology patient responders, with 326 survey invitations sent out. Of the 231 patient participants, 103 responders (44.6% of total survey participants) indicated that they had been diagnosed with vasculitis [Takayasu's arteritis, Giant cell arteritis (GCA), ANCA-associated vasculitis, or IgA vasculitis].

Table 1 lists demographic characteristics of vasculitis patients compared to other rheumatic patient respondents. There were no significant differences in gender, age, education level, or employment status. The majority of survey participants were female (63.1% vasculitis; 75.2% other rheumatic conditions) between the ages of 40 and 64 years old (51.4% vasculitis; 55.5% other rheumatic conditions), who had completed post-secondary education (46.0% vasculitis; 60.3% other rheumatic conditions), and employed (66.0% vasculitis; 74.6% other rheumatic conditions). There were no significant differences in medical history characteristics between the groups.

**Table 1:** Demographic and past medical characteristics of vasculitis patients compared to other rheumatic patients who participated in the COVID-19 vaccine perceptions survey.

**Characteristic**	**Patients with vasculitis Number/total responders (%) *n* = 103**	**Patients with other rheumatic conditions Number/total responders (%) *n* = 128**	***p*-value**
**Demographics**			
**Age**			
18–24	3/103 (2.9)	3/126 (2.3)	0.43
25–39	12/103 (11.6)	21/126 (0.16)	
40–64	53/103 (51.4)	70/126 (55.5)	
>65	35/103 (33.9)	31/126 (24.6)	
Prefer not to say	0 (0.0)	1/126 (0.7)	
**Gender**			
Female	65/103 (63.1)	94/125 (75.2)	0.07
**Education**			
< High school	2/102 (1.9)	3/126 (2.3)	0.16
High school	39/102 (38.2)	33/126 (26.1)	
Post-secondary	47/102 (46.0)	76/126 (60.3)	
Graduate degree	14/102 (13.7)	14/126 (11.1)	
**Household income (Annual)**			
< $69,000	46/103 (44.6)	36/126 (28.5)	**0.03**
>$69,000	41/103 (39.8)	61/126 (48.4)	
Prefer not to say	16/103 (15.5)	29/126 (23.0)	
**Length of diagnosis**			
< 1 year	6/102 (5.8)	17/121 (14.0)	**0.002**
1–5 years	57/102 (55.8)	47/121 (38.8)	
5–10 years	22/102 (21.5)	17/121 (14.0)	
10–20 years	11/102 (10.7)	17/121 (14.0)	
>20 years	6/102 (5.8)	23/121 (19.0)	
**Employment**			
Unemployed	10/103 (9.7)	10/126 (7.9)	0.08
Employed	68/103 (66.0)	94/126 (74.6)	
On disability	19/103 (18.4)	10/126 (7.9)	
Homemaker	6/103 (5.8)	12/126 (9.5)	
**Past medical history**			
**Belief that rheumatic condition was triggered**			
Yes	21/102 (20.5)	40/121 (33.0)	0.10
No	7/102 (6.8)	9/121 (7.4)	
Not sure	74/102 (72.5)	72/121 (59.5)	
**Comorbidities**			
Rheumatic	39/103 (37.8)	36/121 (29.7)	0.20
Other	72/102 (70.5)	80/128 (62.5)	0.19
**Previous severe infection requiring hospitalization**			
Yes	30/102 (29.4)	27/122 (22.1)	0.12
No	59/102 (57.8)	86/122 (70.4)	
Not sure	13/102 (12.7)	9/122 (7.3)	
**COVID-19 vaccine status**			
Vaccinated	81/103 (78.6)	91/126 (81.8)	0.45
Don't want vaccine	11/103 (10.6)	8/126 (6.3)	
Other^*^	11/103 (10.6)	13/126 (10.3)	

* Participants declined to provide their vaccine status or reported being unvaccinated but non-hesitant.

The vasculitis subgroup had a higher proportion of patients with annual household income of CAD 69,000 or less (44.6% vasculitis; 28.5% other), compared to other rheumatologic conditions (*p* = 0.03), with a shorter duration of diagnosed illness (*p* = 0.002), with 55.8% of patients (38.8% other) diagnosed for 1 to 5 years.

At the time of the survey (June–August 2021), 78.6% of vasculitis respondents had received at least one COVID-19 vaccine dose, compared with 81.8% of other rheumatology patients. However, vasculitis patients had non-significantly higher hesitancy rates compared to other participants with other rheumatic conditions (10.6% vasculitis; 6.3% other).

Survey responses from vaccine-hesitant vasculitis patients were further analyzed to identify the factors implicated in a decision to refuse COVID-19 vaccines, in individuals who had never been immunized against SARS-CoV-2, as listed in Tables 2–**4**.

**Table 2:** Vaccine-hesitant vasculitis patients' concerns around COVID-19 and COVID-19 vaccination in patients who participated in the COVID-19 vaccine perceptions survey.

	**Number of patients who responded: number/total responders (% total responders)**			
	**Yes**	**No**	**Not sure**	***P-*** **value**
**COVID-19 infection concerns**				
Rheumatic condition increases risk				
Vaccine-hesitant vasculitis patients	6/10 (60.0)	4/10 (40.0)	0/10 (0.0)	0.15
Other vasculitis patients	63/88 (71.5)	15/88 (17.0)	10/88 (11.3)	
Rheumatic medications increase risk				
Vaccine-hesitant vasculitis patients	3/9 (33.3)	5/9 (55.5)	1/9 (11.1)	0.19
Other vasculitis patients	56/89 (62.9)	25/89 (28.0)	8/89 (8.9)	
Worse outcomes due to rheumatic condition				
Vaccine-hesitant vasculitis patients	5/10 (50.0)	3/10 (30.0)	2/10 (20.0)	0.27
Other vasculitis patients	62/86 (72.0)	11/86 (12.7)	13/86 (15.1)	
Consider self at high risk for getting COVID-19				
Vaccine-hesitant vasculitis patients	5/10 (50.0)	3/10 (30.0)	2/10 (20.0)	0.31
Other vasculitis patients	56/92 (60.8)	30/92 (32.6)	6/92 (6.5)	
**COVID-19 vaccine concerns**				
Speed of development				
Vaccine-hesitant vasculitis patients	8/10 (80.0)	2/10 (20.0)	0 (0.0)	**0.001**
Other vasculitis patients	22/90 (24.4)	56/90 (62.2)	12/90 (13.3)	
Safety				
Vaccine-hesitant vasculitis patients	10/10 (100)	0 (0.0)	0 (0.0)	**0.000**
Other vasculitis patients	28/90 (31.1)	56/90 (62.2)	6/90 (6.6)	
Effectiveness				
Vaccine-hesitant vasculitis patients	7/10 (70.0)	1/10 (10.0)	2/10 (20.0)	0.05
Other vasculitis patients	38/90 (42.2)	44/90 (48.8)	8/90 (8.8)	
Components				
Vaccine-hesitant vasculitis patients	8/10 (80.0)	0 (0.0)	2/10 (20.0)	**0.000**
Other vasculitis patients	19/89 (21.3)	57/89 (64.0)	13/89 (14.6)	
Severe adverse reactions				
Vaccine-hesitant vasculitis patients	9/10 (90.0)	1/10 (10.0)	0 (0.0)	**0.001**
Other vasculitis patients	26/90 (28.8)	58/90 (64.4)	6/90 (6.6)	
Side effects				
Vaccine-hesitant vasculitis patients	7/10 (70.0)	3/10 (30.0)	0 (0.0)	**0.003**
Other vasculitis patients	18/89 (20.2)	67/89 (75.2)	4/89 (4.4)	
Impact on rheumatic condition				
Vaccine-hesitant vasculitis patients	9/10 (90.0)	1/10 (10.0)	0 (0.0)	**0.01**
Other vasculitis patients	38/92 (41.3)	46/92 (50.0)	8/92 (8.6)	
Impact on rheumatic medications				
Vaccine-hesitant vasculitis patients	5/10 (50.0)	3/10 (30.0)	2/10 (20.0)	0.18
Other vasculitis patients	26/91 (28.5)	55/91 (60.4)	10/91 (10.9)	
Risk of blood clots				
Vaccine-hesitant vasculitis patients	7/10 (70.0)	2/10 (20.0)	1/10 (10.0)	**0.04**
Other vasculitis patients	30/92 (32.6)	55/92 (59.7)	7/92 (7.6)	
Getting COVID-19 from vaccine				
Vaccine-hesitant vasculitis patients	5/10 (50.0)	4/10 (40.0)	1/10 (10.0)	**0.000**
Other vasculitis patients	6/89 (6.7)	78/89 (87.6)	5/89 (5.6)	
Know what to do with medications if they get a COVID-19 vaccine				
Vaccine-hesitant vasculitis patients	1/10 (10.0)	6/10 (60.0)	3/10 (30.0)	**0.02**
Other vasculitis patients	39/92 (42.3)	46/92 (50.0)	7/92 (7.6)	

The bold values indicate that the *p*-value is statistically significant using the cut-off of *p* < 0.05.

Table 2 examines concerns related to the impact of rheumatic disease or medications on contracting SARS-CoV-2 or developing worse outcomes, which did not significantly differ between vaccine-hesitant vasculitis and other non-hesitant vasculitis patients. However, vaccine-hesitant vasculitis patients were significantly more concerned about almost every aspect of available COVID-19 vaccines, including safety (*p* < 0.001), components (*p* < 0.001), speed of development (*p* = 0.001), risk of severe adverse reactions (*p* = 0.001), side effects (*p* = 0.003), impact on rheumatology condition (*p* = 0.01), risk of blood clots (*p* = 0.04), and fears that they could develop COVID-19 from COVID-19 vaccines (*p* < 0.001). Vaccine-hesitant vasculitis patients tended to be more concerned about vaccine effectiveness compared to other non-hesitant vasculitis responders, although this was not significantly increased (*p* = 0.05). While vaccine-hesitant vasculitis patients did not significantly differ from other responders regarding concerns about the potential impact of a COVID-19 vaccine on their rheumatology medications (*p* = 0.18), this subgroup was significantly less informed about how to manage rheumatology medications when receiving a COVID-19 vaccine (*p* = 0.02). Additionally, there were no significant differences in responses between vaccine-hesitant vasculitis patients compared to vaccine-hesitant patients with other (non-vasculitis) rheumatic conditions (Supplementary Tables A, B).

Table 3 lists vaccine-hesitant vasculitis patients' perceptions of the government's role in handling the COVID-19 pandemic, showing less approval of the government's COVID-19 response, and concerns that publicly available COVID-19 vaccines were not of the highest quality (*p* = 0.005). There was a trend to believing that the government did not give clear details on available vaccines (*p* = 0.02), less trust of government-provided reports on vaccines (*p* = 0.04) and COVID-19 (*p* = 0.04). Vaccine-hesitant vasculitis patients were like other responders regarding effectiveness of the government's vaccine rollout plan (*p* = 0.40) and the public health measures (*p* = 0.31).

**Table 3:** Vaccine-hesitant vasculitis patients' perceptions on the government during the COVID-19 pandemic in patients who participated in the COVID-19 vaccine perceptions survey.

	**Number of patients who responded: number/total responders (% total responders)**						
	**Completely disagree**	**Disagree**	**Neutral**	**Agree**	**Completely agree**	**N/A**	***P*** **-value**
Government gave clear details on available vaccines							
Vaccine hesitant vasculitis	3/10 (30.0)	2/10 (20.0)	5/10 (50.0)	0 (0.0)	0 (0.0)	0 (0.0)	**0.02**
Other vasculitis patients	7/93 (7.5)	24/93 (25.8)	24/93 (25.8)	28/93 (30.1)	10/93 (10.7)	0 (0.0)	
Trust government reports on details and evidence of vaccines							
Vaccine hesitant vasculitis	4/10 (40.0)	3/10 (30.0)	3/10 (30.0)	0 (0.0)	0 (0.0)	0 (0.0)	**0.04**
Other vasculitis patients	10/93 (10.7)	14/93 (15.0)	30/93 (32.2)	29/93 (31.1)	9/93 (9.6)	1/93 (1.0)	
Believe government acquired highest quality vaccines							
Vaccine hesitant vasculitis	3/10 (30.0)	0 (0.0)	7/10 (70.0)	0 (0.0)	0 (0.0)	0 (0.0)	**0.005**
Other vasculitis patients	4/92 (4.3)	8/92 (8.6)	34/92 (36.9)	35/92 (38.0)	10/92 (10.8)	1/92 (1.0)	
Believe government had effective vaccine roll-out plan							
Vaccine hesitant vasculitis	3/10 (30.0)	2/10 (20.0)	4/10 (40.0)	1/10 (10.0)	0 (0.0)	0 (0.0)	0.40
Other vasculitis patients	10/92 (10.8)	23/92 (25.0)	26/92 (28.2)	23/92 (25.0)	9/92 (9.7)	1/92 (1.0)	
Believe government had effective public health measures							
Vaccine hesitant vasculitis	4/10 (40.0)	1/10 (10.0)	3/10 (30.0)	2/10 (20.0)	0 (0.0)	0 (0.0)	0.31
Other vasculitis patients	14/93 (15.0)	28/93 (30.1)	19/93 (20.4)	24/93 (25.8)	7/93 (7.5)	1/93 (1.0)	
Trust reports on COVID-19 and its spread							
Vaccine hesitant vasculitis	5/10 (50.0)	0 (0.0)	3/10 (30.0)	1/10 (10.0)	1/10 (10.0)	0 (0.0)	**0.04**
Other vasculitis patients	12/93 (12.9)	14/93 (15.0)	19/93 (20.4)	36/93 (38.7)	11/93 (11.8)	1/93 (1.0)	

The bold values indicate that the *p*-value is statistically significant using the cut-off of *p* < 0.05.

Table 4 reports vaccine-hesitant vasculitis patients' views of the healthcare providers involved in their care. Compared to 84.9% of non-hesitant vasculitis responders, only 50% of vaccine-hesitant vasculitis patients indicated having discussed COVID-19 vaccines with their providers (*p* = 0.01), and 40% felt that their healthcare team was able to answer their questions on SARS-CoV-2 vaccines vs. 81.5% of other vasculitis patients (*p* = 0.008). Additionally, these vaccine-hesitant participants were more likely to report that their healthcare providers had no role in their immunization decision-making (100% vaccine-hesitant vs. 8.9% other vasculitis) (*p* < 0.001), had less confidence in the information provided to them by their healthcare team (30% vaccine-hesitant vs. 2.1% other vasculitis) (*p* < 0.001). Only 40% of these patients indicated that they would involve healthcare providers in their immunization decisions, compared to 76.3% other vasculitis (*p* = 0.01), and were much more likely (80% vaccine-hesitant vs. 32.2% other vasculitis) to make their decisions independently (*p* = 0.003). Despite this, vaccine-hesitant vasculitis patients were like other vasculitis responders regarding having spoken to healthcare professionals about COVID-19 vaccine risks and benefits (*p* = 0.08) and being encouraged to get a COVID-19 vaccine (*p* = 0.25).

**Table 4:** Vaccine-hesitant vasculitis patients' perceptions of their healthcare providers in patients who participated in the COVID-19 vaccine perceptions survey.

	**Number of patients who responded: number/total responders (% total responders)**			
	**Yes**	**No**	**Not sure**	***P*** **-value**
**Health care provider perceptions**				
**Spoken to healthcare provider(s) about getting a COVID-19 vaccine**				
Vaccine hesitant vasculitis patients	5/10 (50.0)	5/10 (50.0)	0 (0.0)	**0.01**
Other vasculitis patients	79/93 (84.9)	13/93 (13.9)	1/93 (1.0)	
**Can providers answer your questions regarding COVID-19 vaccines**				
Vaccine hesitant vasculitis patients	4/10 (40.0)	4/10 (40.0)	2/10 (20.0)	**0.008**
Other vasculitis patients	75/92 (81.5)	9/92 (9.7)	8/92 (8.6)	
**Spoken to healthcare provider(s) about risks and benefits of COVID-19 vaccines**				
Vaccine hesitant vasculitis patients	5/10 (50.0)	4/10 (40.0)	1/10 (10.0)	0.08
Other vasculitis patients	67/92 (72.8)	24/92 (26.0)	1/92 (1.0)	
**Did healthcare provider(s) encourage you to get a COVID-19 vaccine**				
Vaccine hesitant vasculitis patients	6/10 (60.0)	4/10 (40.0)	0 (0.0)	0.25
Other vasculitis patients	68/93 (73.1)	18/93 (19.3)	7/93 (7.5)	
**How much did your healthcare provider impact your decision on getting COVID-19 vaccine?**				
(Patients who responded “Yes” to “Did your doctor encourage you to get vaccine”)				
Major role				**0.000**
Vaccine hesitant vasculitis patients	0/6 (0.0)			
Other vasculitis patients	36/67 (53.7)			
Minor role				
Vaccine hesitant vasculitis patients	0/10 (0.0)			
Other vasculitis patients	25/67 (37.3)			
No role				
Vaccine hesitant vasculitis patients	6/6 (100)			
Other vasculitis patients	6/67 (8.9)			
**How confident are you in the information given to you by your healthcare providers?**				
Completely confident				**0.000**
Vaccine hesitant vasculitis patients	1/10 (10.0)			
Other vasculitis patients	45/93 (48.3)			
Mostly confident				
Vaccine hesitant vasculitis patients	3/10 (30.0)			
Other vasculitis patients	40/93 (43.0)			
Somewhat confident				
Vaccine hesitant vasculitis patients	3/10 (30.0)			
Other vasculitis patients	6/93 (6.4)			
Not very confident				
Vaccine hesitant vasculitis patients	1/10 (10.0)			
Other vasculitis patients	2/93 (2.1)			
Not very confident at all				
Vaccine hesitant vasculitis patients	2/10 (20.0)			
Other vasculitis patients	0/93 (0.0)			
**Do the following people help you decide to get a vaccine?**				
**Healthcare professional**				
Vaccine hesitant vasculitis patients	4/10 (40.0)	6/10 (60.0)	-	**0.01**
Other vasculitis patients	71/93 (76.3)	22/93 (23.6)	-	
**Family/friends**				
Vaccine hesitant vasculitis patients	0/10 (0.0)	10/10 (100)	-	0.10
Other vasculitis patients	20/93 (21.5)	73/93 (78.4)	-	
**No one—I make my own decisions**				
Vaccine hesitant vasculitis patients	8/10 (80.0)	2/10 (20.0)	-	**0.003**
Other vasculitis patients	30/93 (32.2)	63/93 (67.7)	-	
**Other**				
Vaccine hesitant vasculitis patients	0/10 (0.0)	10/10 (100)	-	0.64
Other vasculitis patients	2/93 (2.1)	91/93 (97.8)	-	
**Not applicable**				
Vaccine hesitant vasculitis patients	0/10 (0.0)	10/10 (100)	-	0.64
Other vasculitis patients	2/93 (2.1)	91/93 (97.8)	-	

The bold values indicate that the *p*-value is statistically significant using the cut-off of *p* < 0.05.

## 4 Discussion

This study identified factors that vaccine-hesitant vasculitis patients indicated influenced their decision-making around COVID-19 vaccination. To our knowledge, this is the first study specifically analyzing COVID-19 vaccine perceptions in vasculitis patients, a group identified to have increased COVID-19-related concerns and harmful health-related behavior (10), in the context of increased risk of poor COVID-19 outcomes (30).

This survey was administered between June and August 2021, between the third and fourth waves of the COVID-19 pandemic. COVID-19 vaccines were publicly available starting early 2021, with second doses widely accessible in June 2021. At the time of the survey, 78.6% of vasculitis patients had received at least one dose of an approved COVID-19 vaccine, compared with 81.8% of other rheumatology patients. However, slightly more vasculitis patients (10.6%) refused vaccination compared to other rheumatology patients (6.3%), although this was not significantly increased. Similarly, a previous study demonstrated that SARS CoV-2 vaccine acceptance was not significantly different in a subgroup of vasculitis and SLE patients compared to other rheumatic conditions (31).

In this study, vasculitis participants were more likely to have a lower annual household income. Previous research has shown that lower income is associated with increased COVID-19 vaccine hesitancy (32, 33). Additionally, vasculitis patients had shorter length of rheumatic disease diagnosis compared to other rheumatology responders. Although further studies are required to specifically determine if length of disease diagnosis impacts SARS CoV-2 immunization decision-making, a study investigating COVID-19 vaccine acceptance in Takayasu's arteritis patients showed that vaccinated individuals were more likely to have longer disease duration (34).

An earlier report from this dataset demonstrated that rheumatology patients have disease-specific factors that influence their COVID-19 vaccine decision-making, including concerns around vaccine adverse effects, efficacy, and risk of contracting SARS CoV-2 from a COVID-19 vaccine (20). Analysis of the vaccine-hesitant vasculitis patients' responses revealed similar concerns, along with three additional major themes potentially influencing their COVID-19 vaccine refusal. These included significantly greater concerns around COVID-19 vaccines, unfavorable perceptions of healthcare providers, and negative views of the government's role in the COVID-19 pandemic.

Vaccine-hesitant vasculitis patients were significantly more concerned about almost every aspect of COVID-19 vaccines. Additionally, similar to previous studies in rheumatology patients, vaccine-hesitant vasculitis patients were concerned about safety (35), side effects (36), risk of severe adverse events, and risk of thrombosis (35). Previous studies demonstrated that fears around speed of development, safety, and severe adverse events are significant in contributing to vaccine hesitancy (37–39). Vaccine-hesitant vasculitis patients also expressed greater concerns around COVID-19 vaccines' impact on rheumatic condition, which could be related to fears around potential disease flare after exposure to vaccine components (35). Patients can be advised that there are rare case reports of COVID-19 vaccine—exacerbated vasculitis flare (40), although the evidence is limited and does not consistently demonstrate that SARS-CoV-2 immunization induces vasculitis (17).

Vaccine-hesitant vasculitis patients were also less confident and less likely to involve their healthcare providers in COVID-19 vaccine decision-making compared to other responders, and reported feeling that their medical teams were unable to answer their COVID-19 vaccine-related questions. These negative perceptions could be furthering mistrust in healthcare providers and the healthcare system and contributing to vaccine hesitancy. Previous studies have demonstrated that healthcare teams are significant in promoting vaccine acceptance (27–29), and increased willingness to get a SARS-CoV-2 vaccine, if recommended by their doctor (41, 42). Additionally, rheumatology patients have disease-specific concerns (20), which can also be better addressed and managed with healthcare provider support. Given that vaccine-hesitant vasculitis patients in this study were significantly more likely to make their vaccine decisions independently, one intervention may be to provide these individuals with accurate, accessible information after a clinic visit for independent decision-making.

Healthcare providers should counsel patients that approved COVID-19 vaccines are effective, safe, and recommended in rheumatology patients, although a potential for disease exacerbation exists (43, 44). Specifically, patients should be reminded that current evidence does not demonstrate consistently increased risk of disease flare after SARS-CoV-2 immunization, but patients with active disease may be at greater risk for exacerbation (44). Additionally, most disease flares post-COVID-19 vaccination are mild, requiring minimal treatment changes (44). Patients should be informed that particular immunosuppressive therapies used in rheumatic disease management reduce the immune response to vaccination, but vaccination protection is still beneficial and important in disease management (43). Therefore, medication management in the context of SARS CoV-2 immunization should be discussed with rheumatology patients, especially since this study demonstrated that vaccine-hesitant vasculitis patients were less likely to know what to do with their medication when receiving a COVID-19 vaccine. Surprisingly, vaccine-hesitant patients reported being more concerned around the possibility of developing COVID-19 from an approved vaccine, so patients should be informed that approved COVID-19 vaccines do not contain live virus and cannot lead to the development of a full SARS-CoV-2 infection (45).

Vaccine-hesitant vasculitis patients reported being more displeased and less trusting of the government's COVID-19 pandemic role, compared to other responders, which could contribute to vaccine-hesitancy, as trust in the overall health system is critical to vaccine acceptance (46). Additionally, vaccine-hesitant patients found government-provided vaccine information to be unclear, further undermining patient confidence in vaccination decision-making. These patients also reported concerns around the quality of government-acquired COVID-19 vaccines, possibly due to concerns about nearly every aspect of available vaccines, combined with their mistrust in the government's response, and/or potential exposure to vaccine misinformation. Overall, our survey results demonstrate a clear confidence gap between vaccine-hesitant vasculitis patients and the government. Ultimately, this study promotes better understanding of the concerns of a vulnerable patient population, known to have increased COVID-19 related concerns and harmful health-related behaviors, in a background of elevated risk for SARS CoV-2 (10).

### 4.1 Limitations

This study had the limitations inherent in the cross-sectional design and self-reported survey methods (47). Data captured in this study considers a specific timeframe (i.e., between June and August 2021), and is not generalizable beyond that period. Additionally, survey responses are self-reported by voluntary participants, and could be influenced by personal biases, recollection errors, or misunderstanding questions (48). The in-clinic convenience sample of patients is also a limitation, because only rheumatology patients seen in-clinic at that time were invited to complete the survey. Additionally, survey participation required internet access, and computer literacy, which could limit representation of disadvantaged populations. Despite these limitations, the study had a 70.9% response rate (*n* = 231) over a two-month period between the third and fourth waves of COVID-19 (i.e., between June and August 2021).

## 5 Conclusion

This study demonstrated that vaccine-hesitant patients may have multiple themes (e.g., SARS CoV-2 vaccine concerns, unfavorable perceptions of the healthcare system) implicated in their decision to refuse COVID-19 vaccination. Therefore, it is crucial that healthcare providers initiate patient-centered discussions around SARS CoV-2 immunization to help support educated decision-making. Attempts should be made to bridge the confidence gap between vaccine-hesitant patients and healthcare teams through open, respectful and transparent conversation around the evidence, as well as risks and benefits of vaccination, especially as it relates to the patient's particular rheumatic condition and disease management status. Additionally, further studies investigating perceptions in vasculitis patients are needed.

## Data availability statement

The raw data supporting the conclusions of this article will be made available by the authors, without undue reservation.

## Ethics statement

This study received ethics approval from the Health Research Ethics Board at the University of Alberta (Pro00108774). The studies were conducted in accordance with the local legislation and institutional requirements. Written informed consent for participation was not required from the participants or the participants' legal guardians/next of kin because potential participants were informed of the research study and its purpose when they attended a scheduled doctor's appointment. Interested patients voluntarily provided their email address and were subsequently forwarded a link to an anonymous online survey. Participants were informed that they could respond to some or all questions and could also withdraw entirely by not submitting the survey, and participation and submission of the survey was considered implied consent.

## Author contributions

IB: Conceptualization, Formal analysis, Investigation, Methodology, Project administration, Writing – original draft, Writing – review & editing. CE: Data curation, Formal analysis, Writing – review & editing. KK: Investigation, Methodology, Writing – review & editing. LS: Formal analysis, Supervision, Validation, Writing – review & editing. AC: Formal analysis, Writing – review & editing, Validation. DR: Data curation, Methodology, Writing – review & editing. JC: Formal analysis, Methodology, Supervision, Validation, Writing – review & editing. EY: Conceptualization, Formal analysis, Investigation, Methodology, Supervision, Validation, Writing – original draft, Writing – review & editing.
