# Supplementary material for: Understanding COVID-19 vaccine hesitancy in vasculitis patients
Source: Front Public Health. 2023 Dec 4;11:1301492. doi: 10.3389/fpubh.2023.1301492 (PMC10726054; doi:10.3389/fpubh.2023.1301492)
Supplement: Supplementary file 1 [file Table_1.DOCX]

**Table A:** Vaccine-hesitant rheumatic disease patients’ (other than vasculitis patients) concerns around COVID-19 and COVID-19 vaccination in patients who participated in the COVID-19 Vaccine Perceptions Survey.

|  | **Number of patients who responded:**  **Number /total responders**  **(% total responders)** | | |  |
| --- | --- | --- | --- | --- |
|  | **Yes** | **No** | **Not Sure** | **P - Value** |
| **COVID-19 infection concerns** | | | | |
| Rheumatic condition increases risk | | | | |
| *Vaccine-hesitant patients* | 2/7  (28.5) | 3/7  (42.8) | 2/7  (28.5) | 0.59 |
| *Other rheumatic disease patients* | 53/112  (47.3) | 39/112  (34.8) | 20/112  (17.8) |  |
| Rheumatic medications increase risk | | | | |
| *Vaccine-hesitant patients* | 0/6  (0.0) | 4/6  (66.6) | 2/6  (33.3) | 0.15 |
| *Other rheumatic disease patients* | 43/114  (37.7) | 52/114  (45.6) | 19/114  (16.6) |  |
| Worse outcomes due to rheumatic condition | | | | |
| *Vaccine-hesitant patients* | 2/7  (28.5) | 3/7  (42.8) | 2/7  (28.5) | 0.47 |
| *Other rheumatic disease patients* | 58/112  (51.7) | 35/112  (31.2) | 19/114  (16.6) |  |
| Consider self at high risk for getting COVID-19 | | | | |
| *Vaccine-hesitant patients* | 1/7  (14.2) | 6/7  (85.7) | 0/7  (0.0) | 0.20 |
| *Other rheumatic disease patients* | 51/119  (42.8) | 61/119  (51.2) | 7/119  (5.8) |  |
| **COVID-19 vaccine concerns** | | | | |
| Speed of development | | | | |
| *Vaccine-hesitant patients* | 6/7  (85.7) | 1/7  (14.2) | 0/7  (0.0) | **0.01** |
| *Other rheumatic disease patients* | 35/108  (32.4) | 65/108  (60.1) | 8/108  (7.4) |  |
| Safety | | | | |
| *Vaccine-hesitant patients* | 6/7  (85.7) | 1/7  (14.2) | 0/7  (0.0) | **0.04** |
| *Other rheumatic disease patients* | 41/109  (37.6) | 59/109  (54.1) | 9/109  (8.2) |  |
| Effectiveness | | | | |
| *Vaccine-hesitant patients* | 6/7  (85.7) | 1/7  (14.2) | 0/7  (0.0) | 0.08 |
| *Other rheumatic disease patients* | 46/108  (42.5) | 57/108  (52.7) | 5/108  (4.6) |  |
| Components | | | | |
| *Vaccine-hesitant patients* | 6/7  (85.7) | 1/7  (14.2) | 0/7  (0.0) | **0.000** |
| *Other rheumatic disease patients* | 20/105  (19.0) | 70/105  (66.6) | 15/105  (14.2) |  |
| Severe adverse reactions | | | | |
| *Vaccine-hesitant patients* | 6/7  (85.7) | 1/7  (14.2) | 0/7  (0.0) | **0.01** |
| *Other rheumatic disease patients* | 35/108  (32.4) | 68/108  (62.9) | 5/108  (4.6) |  |
| Side effects | | | | |
| *Vaccine-hesitant patients* | 5/7  (71.4) | 2/7  (28.5) | 0/7  (0.0) | 0.18 |
| *Other rheumatic disease patients* | 39/106  (36.7) | 63/106  (59.4) | 4/106  (3.7) |  |
| Impact on rheumatic condition | | | | |
| *Vaccine-hesitant patients* | 6/7  (85.7) | 1/7  (14.2) | 0/7  (0.0) | 0.07 |
| *Other rheumatic disease patients* | 46/110  (41.8) | 57/110  (51.8) | 7/110  (6.3) |  |
| Impact on rheumatic medications | | | | |
| *Vaccine-hesitant patients* | 3/6  (50.0) | 3/6  (50.0) | 0/6  (0.0) | 0.27 |
| *Other rheumatic disease patients* | 24/106  (22.6) | 72/106  (67.9) | 10/106  (9.4) |  |
| Risk of blood clots | | | | |
| *Vaccine-hesitant patients* | 6/7  (85.7) | 1/7  (14.2) | 0/7  (0.0) | 0.07 |
| *Other rheumatic disease patients* | 42/105  (40.0) | 56/105  (53.3) | 7/105  (6.6) |  |
| Getting COVID-19 from vaccine | | | | |
| *Vaccine-hesitant patients* | 4/7  (57.1) | 2/7  (28.5) | 1/7  (14.2) | **0.000** |
| *Other rheumatic disease patients* | 5/101  (4.9) | 89/101  (88.1) | 7/101  (6.9) |  |
| Know what to do with medications if they get a COVID-19 vaccine | | | | |
| *Vaccine-hesitant patients* | 0/6  (0.0) | 4/6  (66.6) | 2/6  (33.3) | 0.17 |
| *Other rheumatic disease patients* | 41/109  (37.6) | 45/109  (41.2) | 23/105  (21.9) |  |

**Table B:** Vaccine-hesitant vasculitis and other vaccine-hesitant rheumatic disease patients’ concerns around COVID-19 and COVID-19 vaccination in patients who participated in the COVID-19 Vaccine Perceptions Survey.

|  | **Number of patients who responded:**  **Number /total responders**  **(% total responders)** | | |  |
| --- | --- | --- | --- | --- |
|  | **Yes** | **No** | **Not Sure** | **P – value** |
| **COVID-19 infection concerns** | | | | |
| Rheumatic condition increases risk | | | | |
| *Vaccine-hesitant vasculitis patients* | 6/10  (60.0) | 4/10  (40.0) | 0/10  (0.0) | 0.15 |
| *Vaccine-hesitant rheumatic disease patients* | 2/7  (28.5) | 2/7  (28.5) | 3/7  (42.8) |  |
| Rheumatic medications increase risk | | | | |
| *Vaccine-hesitant vasculitis patients* | 3/9  (33.3) | 5/9  (55.5) | 1/9  (11.1) | 0.22 |
| *Vaccine-hesitant rheumatic disease patients* | 0/6  (0.0) | 4/6  (66.6) | 2/6  (33.3) |  |
| Worse outcomes due to rheumatic condition | | | | |
| *Vaccine-hesitant vasculitis patients* | 5/10  (50.0) | 3/10  (30.0) | 2/10  (20.0) | 0.67 |
| *Vaccine-hesitant rheumatic disease patients* | 2/7  (28.5) | 3/7  (42.8) | 2/7  (28.5) |  |
| Consider self at high risk for getting COVID-19 | | | | |
| *Vaccine-hesitant vasculitis patients* | 5/10  (50.0) | 3/10  (30.0) | 2/10  (20.0) | 0.07 |
| *Vaccine-hesitant rheumatic disease patients* | 1/7  (14.2) | 6/7  (85.7) | 0/7  (0.0) |  |
| **COVID-19 vaccine concerns** | | | | |
| Speed of development | | | | |
| *Vaccine-hesitant vasculitis patients* | 8/10  (80.0) | 2/10  (20.0) | 0/10  (0/0) | 0.76 |
| *Vaccine-hesitant rheumatic disease patients* | 6/7  (85.7) | 1/7  (14.2) | 0/7  (0.0) |  |
| Safety | | | | |
| *Vaccine-hesitant vasculitis patients* | 10/10  (100.0) | 0/10  (0.0) | 0/10  (0.0) | 0.21 |
| *Vaccine-hesitant rheumatic disease patients* | 6/7  (85.7) | 1/7  (14.2) | 0/7  (0.0) |  |
| Effectiveness | | | | |
| *Vaccine-hesitant vasculitis patients* | 7/10  (70.0) | 1/10  (10.0) | 2/10  (20.0) | 0.45 |
| *Vaccine-hesitant rheumatic disease patients* | 6/7  (85.7) | 1/7  (14.2) | 0/7  (0.0) |  |
| Components | | | | |
| *Vaccine-hesitant vasculitis patients* | 8/10  (80.0) | 0/10  (0.0) | 2/10  (20.0) | 0.24 |
| *Vaccine-hesitant rheumatic disease patients* | 6/7  (85.7) | 1/7  (14.2) | 0/7  (0.0) |  |
| Severe adverse reactions | | | | |
| *Vaccine-hesitant vasculitis patients* | 9/10  (90.0) | 1/10  (10.0) | 0/10  (0.0) | 0.78 |
| *Vaccine-hesitant rheumatic disease patients* | 6/7  (85.7) | 1/7  (10.0) | 0/10  (0.0) |  |
| Side effects | | | | |
| *Vaccine-hesitant vasculitis patients* | 7/10  (70.0) | 3/10  (30.0) | 0/10  (0.0) | 0.94 |
| *Vaccine-hesitant rheumatic disease patients* | 5/7  (71.4) | 2/7  (28.5) | 0/7  (0.0) |  |
| Impact on rheumatic condition | | | | |
| *Vaccine-hesitant vasculitis patients* | 9/10  (90.0) | 1/10  (10.0) | 0/10  (0.0) | 0.78 |
| *Vaccine-hesitant rheumatic disease patients* | 6/7  (85.7) | 1/7  (14.2) | 0/7  (0.0) |  |
| Impact on rheumatic medications | | | | |
| *Vaccine-hesitant vasculitis patients* | 5/10  (50.0) | 3/10  (30.0) | 2/10  (20.0) | 0.44 |
| *Vaccine-hesitant rheumatic disease patients* | 3/6  (50.0) | 3/6  (50.0) | 0/6  (0.0) |  |
| Risk of blood clots | | | | |
| *Vaccine-hesitant vasculitis patients* | 7/10  (70.0) | 2/10  (20.0) | 1/10  (10.0) | 0.63 |
| *Vaccine-hesitant rheumatic disease patients* | 6/7  (85.7) | 1/7  (14.2) | 0/7  (0.0) |  |
| Getting COVID-19 from vaccine | | | | |
| *Vaccine-hesitant vasculitis patients* | 5/10  (50.0) | 4/10  (40.0) | 1/10  (10.0) | 0.88 |
| *Vaccine-hesitant rheumatic disease patients* | 4/7  (57.1) | 2/7  (28.5) | 1/7  (14.2) |  |
| Know what to do with medications if they get a COVID-19 vaccine | | | | |
| *Vaccine-hesitant vasculitis patients* | 1/10  (10.0) | 6/10  (60.0) | 3/10  (30.0) | 0.72 |
| *Vaccine-hesitant rheumatic disease patients* | 0/6  (0.0) | 4/6  (66.6) | 2/6  (33.3) |  |
